# Health Tract, No. 183

**Published:** 1864-02

**Authors:** 


### HEALTH TRACT, No. 183.

### THE SICK SCHOOL-GIRL.

I KNOW an only daughter of fourteen, sole heiress to a fortune of hundreds of thousands of dollars, the petted child of fond but foolish and misguided parents, in a distant city, and yet she is miserable in mind and body for three fourths of her waking existence. She rides to school, less than a mile away, in a splendid equipage every fair morning; if it is threatening weather, she stays at home. She seldom goes to bed sooner than eleven or twelve o'clock. She is barely ready to start to school any morning sooner than nine. On the mornings of Saturday, Sunday, holidays, and bad days, eleven o'clock still finds her in bed. Her nights are spent at parties, balls, operas, theaters, or in frivolous company. If none of these are available, she reads novels in bed by gas-light for hours and hours together; but an almost invariable custôm, before retiring, especially after returning from the ball or the theater, is to take a hearty supper. The result of this last practice is, that she never has any appetite for breakfast, not much more for dinner, so that the only full meal of the day is just before retiring. The legitimate results of such a training on body, mind, and heart are sadly suggestive. This spoiled child is never well any three days in succession, and has alarming attacks of a dangerous malady a dozen times a year, besides almost daily complaints of headache, tiredness, cold feet, weight or burning at the stomach; and more or less of a "little cold" all the time. To suppose that this child will ever reach the maturity of womanhood is absurdity itself.

The effects which such a mode of life has on the mind are not less baleful. Such a girl can not be "educated" in any single branch of knowledge, in any single accomplishment. The excitement of the nightly novel and theatre will wear out the mental energy before its time, and must as certainly unfit her for the realities and the labors of life as the excitement arising from spirits incapacitates the body for steady, effective labor.

The effects of such a defective training on the heart, the temper, the soul are the highest types of injustice, selfishness, and a sad destitution of human sympathies. These bear hardest and first on the servants. The coachman must remain on his box in the street until midnight, however inclement the weather; her maid must sit up to open the door when she comes home, and the cook the same, in order to prepare her a lunch before retiring. Yet if the cook is not up at daylight to prepare the regular family breakfast, and the maid to open the house and sweep the halls and stoop and pavement in time for the earliest visitor, and the coachman to take his master to 'Change, or his mistress to her early shopping, they are all subject to the severest reprimands, with the apparently unanswerable question: "Are you not paid to wait on me?" The laundress also suffers, for miss takes not the slightest pains to "save her clothing," either from being soiled, or torn, or disordered, on the plea that "she is hired to wash, and we pay her for it." The intelligent and generous mind sees at once the absurdity of such views, their injustice and their stony-heartedness. No one has a right to make one hand's turn of unnecessary labor for the meanest scullion of the kitchen, nor to demand unseasonable labor or service; every servant in every humane family has a right to all the sleep that can be taken, and an equal right to demand regularity in all the movements of the household, or an extra compensation for the lack of it. We should demand nothing which costs another unnecessary trouble or pain.

